# Book Reviews

**Published:** 1810-06

**Authors:** 


### CRITICAL ANALYSIS

OF THE

### RECENT PUBLICATIONS

IN THE

### DIFFERENT BRANCHES OF PHYSIC, SURGERY, AND MEDICAL PHILOSOPHY.

---

*A Treatise on Cheltenham Waters, and Bilious Diseases; second Edition, newly arranged, with numerous Additions, and Two Plates.* By Thomas Jameson, M. D. of the Colleges of Physicians in London and Edinburgh; formerly Physician and Lecturer at the Finsbury Dispensary, London; now Resident Physician at Cheltenham. 8vo. pp. 219.

When a physician resident in a watering place sits down to write a Treatise upon the Medicinal Waters of that place, we may fairly anticipate a recommendation of their virtues in the cure of various diseases; for unless a person so circumstanced, could speak well of them, he would undoubtedly be silent; even the author's declaration, that his "having *now* no connection with any well, nor any predilection for one of the wells more than another," does not completely convince us that "his opinion concerning these waters *must* be completely unbiased by any interested motives," for the resort of patients to any of the wells, is, we presume, a source of emolument to him, more especially if they are impressed with a due sense of the importance of the advice Dr. Jameson has given them in the end of his preface.

"Prudence

"Prudence requires that invalids should always be directed, before they drink the water, whether they are to pursue the laxative or purging plan, and what kind of water is best suited to their case. And after they have drank them a certain time, it would be proper to ascertain with accuracy, whether changes have not taken place in their constitution, or their disease, to interdict the further use of the waters."

Whether *a popular work* on the medical qualities of Cheltenham waters, and containing an enumeration of diseases to which they are adapted, was requisite, or how far it may be useful, will admit of some doubt; for there are few patients at a distance, whose circumstances will enable them to avail themselves of the benefit of these waters, who do not employ some medical practitioner, whose advice as to the waters being adapted to their disease, must at least be equal to what they can obtain from any popular treatise whatever. If Dr. J. claims the right of prescribing to patients *how* to drink the waters when at the springs, he should certainly leave to other practitioners the exercise of their judgment when to send them thither. Whatever information Dr. J. can impart to the medical profession on this subject, whether to correct any erroneous statement they may have received of the analysis of these springs, or to add to the knowledge of their effects on the human system, and thereby to guide their judgment in prescribing them, his brethren would be thankful for; and as we can only consider his treatise valuable in proportion as it effects either of these objects, we shall proceed to extract briefly the substance of his experience on these points.

The author divides the springs of this town into two classes, the aperient saline wells, and the simple carbonated chalybeate wells; these are again subdivided according to the degree of impregnation and the relative proportion of their ingredients; "they all supply waters with one leading feature, although there is some variety of character in every one of them." It is unnecessary to enter into minute particulars on this head, since the author assents to the accuracy of those analyses which have already been published; the last of which, Mr. Accum's, he quotes for the contents of some of the waters. This part of the work we cannot abridge, and have nothing to object to, since the experiments are detailed with accuracy, and the results given faithfully; it will be sufficient to state, that generally, the saline properties are owing to the solution of sulphate of magnesia and sulphate of soda, to which is frequently added a considerable proportion of muriate of soda, "but the operation on the bowels is nearly the same in all." They almost all possess more or less iron, and some contain it in large proportion. "Sulphurated hydrogen gas is also contained in most of them, notwithstanding they hold iron in solution." After the description of the wells, and an account of their chemical and medical qualities, we have a chapter on the modes of administering the different kinds of waters. This contains some general directions which may be be useful, as to the season of the year in which they are most efficacious, the time of day for drinking them, the dose and temperature, the duration of the course, and the mode of discontinuing their use; upon all of these heads, we must refer our readers to the work itself, and pass over to the author's account of diseases, wherein the waters are indicated, and those wherein they are prejudicial, as affording us an opportunity of examining some of his opinions, and ascertaining how far they are agreeable to the present generally received doctrines of medicine, or are supported by experience. This, however, cannot be done without some difficulty, for in general his propositions are stated so loosely, and his terms are employed so inaccurately, that we are not always certain we exactly understand the author in the sense he means to express himself. Thus, for instance, when he tells us that a gradual and constant application of intense heat to the human body induces *relaxation*, and that iron *braces* the stomach, and is useful in diseases which require a *bracing* remedy, we are at first apprehensive that he is estimating the chemical operation of substances on the *dead* animal fibre, unmindful of the laws of organic life, instead of reporting the changes he has observed to be produced in health or disease by these different agents. We shall, notwithstanding, endeavour to separate facts from hypotheses, and inform our readers of the most valuable results of the author's experience.

According to him, then, the principal diseases which require a course of purging waters, are the following:

"Inflamed and schirrous liver or spleen. Torpid action of the liver. Bilious state of the stomach. Habitual costiveness. Hypochondriacal complaints. Sick head-ach with bilious vomitings. Some kinds of bilious purgings. Jaundice and biliary concretions. Depraved appetite and indigestion. Pimply eruptions, called scurvies. Scaly, and scurfy states of the skin. Inflammations of the skin of the face. Exudations, and watery humours of the skin. Some kinds of scrofulous tumours. Inflammations of the eyes and eyelids. Inflamed ulcers, and discharges of the legs. Some stages of rheumatism and gout. Inflammatory asthma. Female diseases. Piles and fistula. Diseases of the kidneys, gravel, and stone. Intestinal worms."

It is impossible to reduce this enumeration to any nosological arrangement whatever, many of these *diseases* being merely symptoms, and some of their names unintelligible. What the author means by a bilious state of the stomach, we are at a loss to ascertain; in vain we search the nosological synopses, we no where find such a disease; we are equally unsuccessful in our examination of practical writers, in no history of any disease can we find such a symptom mentioned. It is then founded, perhaps, upon some fanciful hypothesis; we know, indeed, that *bilious diseases* is now the popular phrase, and has entirely superseded *nervous complaints* among fashionable valetudinarians; but we think too highly of Dr. J. to imagine he would countenance or propagate unfounded popular lar errors, or wish to ingratiate himself into the good graces of his patients, by adopting their nonsensical whims; a trick of charlatanry unworthy a man of character, and a member of so liberal a profession as medicine. The few remarks that are given by the author, on each of these states of disease, are not very important, and we would rather confide in his experience of the utility of Cheltenham waters in all of them, than enter into a refutation of the hypotheses upon which he explains their action. The purging waters are contraindicated in cases of debility from old age, and in some diseases, the list of which is thus introduced by the author.

"I *consider* four or five motions a day, produced by any kind of purging remedy for eight days together, to lessen the lymphatic part of the blood as much as the loss of half a pint of blood from the arm would do in the same space of time. The impropriety, *therefore*, of using purging saline waters in the following diseases, must be perfectly obvious:

"Nervous diseases. Palsies. Consumptions. Hæmorrhages. Dropsies. Fevers, and very acute diseases."

We do not admit the conclusion to be a necessary induction from the premises; and it is not obvious to us, but that in some states of these diseases, the purging waters may be used with advantage; and notwithstanding consumption is included in this list, "the author has found that it may be saf ly employed in some cases of tuberculated lungs, and is extremely useful in many cases of dry cough, in promoting expectoration, by dilution, and keeping the body cool by its saline impregnation."

Their use is ambiguous, Dr. Jameson says, in affections of the head, not because purging is not very serviceable in most diseases of the head, but on account of over distension of the stomach produced by a large quantity of water, as our author justly remarks: to obviate this, he recommends the purgative effect to be quickened by an additional quantity of salts, and asserts that Cheltenham water, when it purges, has a tendency *to cool the brain*, and to lessen plethora in the head."

Steel waters are indicated in chronic diseases accompanied with debility, and unattended with feverish symptoms; they may be alternated with the purging waters, or even drank on the same day with advantage: they are contraindicated in inflammatory diseases, visceral obstructions, determinations of blood to the head, in dropsies, and in calculous diseases

"All carbonated chalybeate waters are more invigorating in proportion to the iron they contain, than is observable from any artificial preparation of the metal. An eighth part of a grain of iron, contained in a dose of steel water, having more salutary effects on the constitution, than two or three grains of the oxid of iron in powder. The reason is, the acid of the stomach dissolves but a small part of ferruginous powder, and the remaining portion passes downwards without entering the circulation, and thereby gives the alvine evacuations a dark colour. Whereas repeated

 doses doses of chalybeate waters do not depend upon the fluids of the stomach for solution, and seldom produce the same effects upon the contents of the alimentary canal."

This *reason* does not appear to us perfectly satisfactory, although we readily admit the fact; it is probable, that out of two or three grains of oxid of iron, $\frac{1}{8}$ of a grain is dissolved in the stomach; the difference, therefore, depends upon some other circumstance, and may be thus accounted for; the iron, when not largely diluted, acts as a stimulus upon the *stomach* itself, and produces indirect debility, which counteracts the tonic effect of the metal; whereas, by large dilution, the tonic effect is secured without the stimulant one, as is exemplified in the use of port wine in hœmorrhage; given alone, it stimulates the vessels and increases the hæmorrhage; when mixed with water, it acts as a restringent, and checks the bleeding. In practice, accordingly, we find much more beneficial effects to arise from exhibiting the tincture of the salts of iron in a large dose of water, than when a single wine glass full only is employed; at the same time we must remember that the superior virtues of the chalybeate waters drank at the wells, depend in no inconsiderable degree upon several concomitant circumstances affecting the patient, as company, amusement, &c.

We come now to what the Author considers a very important part of his volume, for in his Preface he thus speaks of it.

"The chapter on bilious diseases has been greatly enlarged, from the many opportunities the author has lately had of observing the character they generally assume in Britain. It was not without regret, that he found himself under the necessity of treating their history more medically than is usual in popular works, but he could not neglect the favourable opportunity of communicating his sentiments upon a subject, which has become of the utmost importance in modern times."

What has rendered this subject of more importance in modern times we do not know; that bilious diseases are said to be now more prevalent, arises chiefly from the vague employment of the term, and we are sorry that the author has not been more accurate in his definitions, as this part was intended for medical Readers. In the first paragraph we are told that half the invalids who visit Cheltenham are affected with *bilious* disorders; in the second, that *biliary* diseases spontaneously divide themselves into two principal classes. These are not convertible terms, although our author has so employed them; biliary diseases, strictly speaking, mean those which depend upon an altered structure or deranged functions in the liver or its appendages; bilious diseases, those in which bile is contained in some of the secretions or excretions of the body. Cholera in its mildest and most simple state being merely an increased flow of bile into the intestines, in which neither the structure nor functions of the liver are *impaired*, is an example of the latter; torpor or paralysis of the liver, which is here said to be one one of the most frequent *bilious* diseases met with in this country, is undoubtedly a *biliary* disease, and a fair instance of the former.

We proceed then to give a short account of the different states of these diseases, in which Cheltenham waters are likely to prove serviceable, without following our author minutely through his arrangements or descriptions, which are neither scientific nor satisfactory. Chronic inflammation of the liver, torpor, or paralysis of the liver, and congestion of fluids in that organ, whether sanguineous, serous or lymphatic, are all states in which these waters are indicated; to the use of which should be joined exercise in the open air to invigorate the habit, which will promote the absorption of fluid, or indurated matter in the substance of the organ. But the most numerous class of diseases in which Cheltenham waters may be used with advantage, is what our author has denominated "diseases with a deranged state of the bile," comprehending such as arise either from the superabundace, deficiency, obstruction, or deficiency of that fluid. As to the general causes of these various affections, we are informed, that "most bilious diseases proceed from colonial heat, and when generated in cold climates, they arise from a peculiar temperament of body, from getting cold, from intemperance in eating and drinking, and from irregular modes of life. But they are most generally derived from the burning heat of the solar rays, or the liquid fire of the still, which is the reason that men are more frequently affected with these complaints than women."

The theory of the operation of these causes is thus set forth:

"A gradual and constant application of intense heat to the human body, imperceptibly changes the state of its stamina, by inducing relaxation and debility, which predispose the habit to putrid and bilious diseases. Thus a greater external circulation of blood, and increased discharge from the cuticular pores, are general conditions of the system in hot climates; observable by an increased sensibility of the skin to external cold, and by a constant moist state of the surface, requiring change of linen once or twice a day. This exterior circulation exhausts and weakens the interior vessels of the body, from which vital energy and strength are chiefly derived. While at the same time, the increased circulation of blood in the liver, from heat, augments the secretion of bile, by which means it is transmitted more copiously to the alimentary organs in a thin and crude state, so as to irritate them, particularly in their excited state. But tropical diseases seldom occur, even in the predisposed state of the body, until a morbid irritability, or an erysipelatous inflammation has taken place in the internal membranes of the chylopoetic viscera, which subjects them to receive supernatural stimulus from their own fluid, in a manner similar to what happens in catarrh, with the mucous membrane of the throat, when inflamed; the disease is increased and propagated by the irritation it receives from its own secretion, which differs only from its natural state in quantity and increased tenuity."

As

As to their effects upon the system at large, we learn, that "So great is the influence of the liver over the whole system, that there is scarcely a part which does not sympathize with its affections, and many complaints referred to other organs, take origin in this gland. Gout, apoplexy, *hypochondria*, and piles, are frequently associated with diseased liver; and from the necessity of the biliary secretion for the healthy actions of the intestines and stomach, the hepatic and alimentary organs reciprocally partake of each other's diseases."

The last part of the chapter contains some remarks upon vitiated bile, and also states the principles upon which the author explains the beneficial effects of the Cheltenham saline waters. —"Without entering into the controversy, concerning bile as a cause or symptom of diseases, we can freely state our complete evidence of its existence in a vitiated state, in a great number of instances. In dyspepsia, bile is often thrown up with the contents of the stomach in a highly acrid and corrosive state. Bile is vomited in cholera morbus evidently diseased, and sometimes with as great rapidity as if a patient had swallowed poison. Copious discharges of fœtid and putrid bile are not uncommon occurrences in fevers of the putrid, bilious, and remittent kinds, as well as in the plague, colics, diarrhœa, and dysentery.

"The opinion of the ancients concerning the destructive properties of black bile, was greatly confirmed by the utility of purgative medicines in the cure of malignant diseases; and there is no doubt but the saline waters of Cheltenham are of great service in removing acrid and vitiated bile from the alimentary canal, while at the same time they increase the demand of the system for new matter, to secrete healthier fluids. Upon these principles, we explain many of their excellent effects in most bilious diseases of a chronic nature."

The remainder of the volume is occupied in the description of the baths and different kinds of bathing at Cheltenham; this part of it, although interesting to the visitor who resorts thither, affords nothing on which we can offer any remark. We can only say in conclusion, that we wish the volume had been written with more care, that its language had been less incorrect, and freed from its numerous orthographical and grammatical errors, especially as it was intended for the general Reader, who from this specimen must form rather an indifferent opinion of the literary acquirements of the members of the medical profession in general.

---

*Tabular Views of the Anatomy of the Human Body.* By HENRY H. ASHFORD, M. D. Member of the Royal College of Surgeons in London, and Assistant Surgeon in the Royal Artillery. London, printed for the Author.

THE Author has not given us a word of Preface or Introduction explanatory of his intentions in publishing these tabular views, nor informed us for whose use they are principally designed. Convinced as we are of the absolute necessity of frequent and various dissection dissection to constitute the practical anatomist, and even to impart to the medical student that portion of anatomical knowledge it is important to him to acquire, we should not think favorably of any expedient that might induce young men to forego the labours of the dissecting room, yet we do not consider these tables as ill adapted to the use of those practitioners, who having left the schools of science, require some good compendium which they may occasionally consult, and which will recal to their minds in the order they were acquired, those minutiæ of anatomy which so readily slip from the memories of persons who are no longer busily engaged in the actual prosecution of anatomical pursuits.

Looking upon it, therefore, in this point of view, we consider the work before us as a well executed plan, the subjects being well arranged, and the descriptions clear, yet concise. It consists of twenty-three Tables, of which, six are taken up in the description of the bones, two for that of the joints and ligaments, four are appropriated to the muscles; one to the brain and its membranes, two to the nerves, and as many to the organs of sense. The arteries are comprised in two, the veins and lymphatics in one. The viscera occupy two tables; and the work is completed by an account of the organs of generation and urine in the last table. In addition to the utility it will be of to the older practitioner, this book may serve as a complete vade mecum for the dissector, and an useful auxiliary to the candidate for collegiate honours. In describing the muscles, their compound actions are not noticed, and, perhaps, it would have been inconsistent with the intention of an elementary work to have done so. The author appears to have adhered to the usual arrangement, and has not adopted the peculiar one of Dr. Barclay, which is founded entirely upon the consideration of their combined action. However valuable the arrangement of this latter author may be to the physiologist, the method of Albinus and Innes must be acknowledged to offer greater facilities to the mere anatomical student, and has therefore been judiciously employed in the present instance.

Although we are satisfied of the strict propriety of the title of this work, consisting of tables, which give by a single inspection, every circumstance pertaining to the subject described, yet we have some doubt whether it may not occasionally mislead; the term *Tables* being so familiar to the ear of the anatomical student, as conveying an idea of engraved representations, accompanied with explanatory references, nothing of which kind is to be found here, every Table containing letter-press only, but which being of a small type, comprises a great deal of information in the smallest possible compass. Upon the whole, the Tabular Views may be considered as a valuable digest of anatomical knowledge, which while they will facilitate the attainment of that science, will not, we hope, supersede that more important source of improvement, practical dissection, by which alone an accurate acquaintance with the structure and functions of the human body can be acquired.

EDINBURGH

### Edinburgh Journal. No. xxii.

( Concluded from our last, pp. 428—432. )

The author concludes, that the indisposition of the sore itself to heal and the derangement of the general system, arise from the application of the pus secreted by the vessels of the ulcer to the surface of the ulcer itself. Many difficulties oppose themselves to this opinion. What first occasioned the vessels to secrete pus capable of producing these effects upon the surface of the ulcer? and how can the mere removal of that pus, when secreted, alter the disposition or action of those vessels? Some change must take place in the action of the secreting parts, before any alteration can be produced in the matter secreted; this is surely not to be effected by absorbing the pus after it is secreted. The pouring out this noxious matter must have been *preceded* by a certain state or action of the vessels, and as this state was not originally produced by the secreted pus, it may, for ought we can say, be kept up independent of it. We can readily understand that the inflammatory state of the vessels of the part being removed by poultices and fomentations, they may assume a healthy action, and pour out pus devoid of irritating qualities. We can as easily comprehend that if the unhealthy action of the secreting vessels depends upon a morbid constitutional affection, a removal of this may restore the healthy action to the vessels of the part, and the same beneficial consequences may follow. That a state of disease may be increased and extended, by suffering an irritating extraneous substance to remain long on the part is probable, and we would not be understood as objecting to its removal, although we cannot attach the importance to this circumstance which the author has done. "In some cases," he says, "where the constitution was greatly affected, I attended to the local disease only; yet, as a general practice, I should hold it good to pay a proper attention to the constitutional derangement." This general practice seems to be inconsistent with the conclusion he has drawn in a former part of the paper, "that the constitution is affected from the local disease; and as the local disease assumes a healthy action, the constitution will resume its original vigour." We certainly are not convinced by this paper, that any ulcer is to be *cured* by absorbing upon lint the pus secreted from it; the utmost we can allow to this management is, that it may occasionally protect the surrounding sound parts from acrimonious substances, which might otherwise irritate and inflame them. The author himself does not in practice *entirely rely* upon the dry lint, as over it be applied a linen, kept wet with an evaporating lotion, *to allay the surrounding inflammation.*

Article 7.—*Observations on Elephantiasis.* By Arthur Edmondston, M. D. Fellow of the Royal College of Surgeons, Edinburgh.

The Edinburgh Journal, No. xx, for October, 1809, vol. 5, p. 499.

499, contains a letter from Thomas Christie, Esq Medical Superintendant General, to the Editor of the Ceylon Gazette. In this paper, the author has introduced the following note. "Lepra Arabum, or Elephantiasis of the Greeks; for a good description of which, as it appears in Ceylon, see Dr. Adams's Treatise on Morbid Poisons."

After the account of one author, who had examined elephantiasis in a lazar-house, containing thirty subjects in different stages of the disease, and made a report on the subject to the Portuguese government, and after finding this report confirmed by the Medical Superintendant General of Ceylon, from his own observations in a lazar-house in that island, we should not have expected to read that "elephantiasis is known only by name;" nor should we have looked for an accurate history of it so far north as Scotland. However, the paper before us contains many *decisive* remarks on that disease, and also on trichoma and frambœsia; and although Dr. Cullen in his nosology, very modestly informs his readers, that he had never seen either elephantiasis, frambœsia, or trichoma, yet Dr. Edmondston, who seems familiar with them all, tells us that Cullen's description of elephantiasis is unexceptionable. It may be so, but we conceive the illustrious professor would have been better pleased to see his description supported by an official report from Madeira, confirmed by the Medical Superintendant General of Ceylon, than by one who only informs us of what he has seen in the wards of a northern hospital.

ARTICLE 8.—*Observations on the Treatment of the Sick returned from Corunna.* By B. G. B.

THE observations of this author were suggested by reading Mr. Hooper's paper in the Edinburgh Journal for October last, and apply principally to the case of Mr. Williams, detailed in that paper. He entertains little doubt of the fever being originally of an inflammatory nature, and that the brain was very powerfully affected. On this ground, he reprobates the treatment that was adopted, of committing his chance of recovery to calomel alone, for the first three days, and suffering him to remain all that time without any evacuation per anum. Had copious bleeding been instituted in the first instance, he thinks there is little doubt but it would have so far relieved the brain immediately, as to have allayed the disordered action of the stomach also, and thereby have afforded an opportunity of giving such purgatives as would still further have tended to reduce the fever.

The author highly approves of bleeding and evacuations in cases of fever, where there is great affection of the head; and he recommends, when the fever is so violent, as not to give way after three or four copious bleedings from the arm, and the strength still allows a further loss of blood, that it should be taken from the jugular vein; a practice he has found very beneficial.

"Indeed, (says he) until medical men in general are less alarmed about debility, I shall despair of ever seeing a rational treatment of either either fever or inflammation; for the practice of what is called throwing in bark, and giving stimulants, to obviate debility in such cases, has been hitherto so universal, and so destructive, that a more judicious practice will be many years in debt, before it shall have made a sufficient compensation to the public for the disasters of the former."

That bleeding must be highly serviceable in inflammatory affections of the brain, there can be no doubt; but until such are ascertained to be invariably the proximate cause of fever, some caution is necessary, lest we interrupt the natural progress of cure in fever by too much attention to one of its symptoms only. It may frequently, by removing the congestion of the vessels in the brain, be serviceable in obviating a source of danger during the continuance of the fever, when it may be utterly incapable of removing the fever itself. We perfectly concur with the author in the propriety of the following remark.

"Medical men, in the army more particularly, should also consider, that the subjects of fever with them are young men, and men in the greatest vigour of life, when they are more likely to be of an inflammatory habit; consequently, there should be more anxiety to counteract this at first, than to obviate debility, to which, in the first instance, there is generally the least disposition."

Article 9. — *Case of Tetanus, with Appearances on Dissection. Translated from* Stoll, *with Observations, by a Naval Surgeon.*

After four days continuance of tetanic spasms and occasional attacks of epileptic convulsions, during which time nothing appears to have been done for the patient but throwing up first an oily and then an irritating injection, and giving him Epsom salts and manna, the subject of this case died. On opening him, some of the intestines, and a great portion of the mesentery, were found very red and livid, with vessels larger and more numerous than usual, which appearances the author does not know whether to attribute to inflammation, *bruise*, or congestion: the jejunum contained worms. The annotator thinks "it would be sinning against the chaste spirit of induction, to conclude from a single case, that the inflamed state of the intestines was the cause of the tetanus." The surgeon of the Naval Hospital at Barbadoes, he tells us, has, in some dissections of subjects who died of tetanus, discovered analogous appearances; he therefore adds, "but when we find the same thing to have been remarked by others, the observation becomes of more importance, and in the light of cause and effect, assumes a higher tone of credibility."

Article 10.—*On the Degree of Importance which should be attached to the Functions of the Uterus in regard to Health.* By Mr. Fogo, Newcastle.

Mr. Fogo commences his paper, by informing us that he has always differed in opinion from medical writers, on the subject of the very great importance of the functions of the uterus; we expected, therefore, therefore, he would have communicated some powerful reasons for his doing so, and we were prepared to examine them with no little degree of attention. We meet, however, with only a few inconclusive arguments, some ineffectual attempts at wit, and some erroneous conclusions, drawn from an imperfect statement of facts. To follow him through the whole of these, would be tedious to ourselves, and uninteresting to our readers; it will be sufficient, briefly to state the philosophic manner in which he infers that the suppression of the menstrual discharge does not influence the health of the individual; that it may be a sign of ill health, but can never be a cause of it.

"The female enjoyed the best health and spirits from birth till she arrived at the age called of puberty, suppose fifteen years. At this period, an effusion of a small quantity of blood took place from the organs of generation, and which returned, at periodical times, till she arrived at another age, say forty-five; the effusion then ceased at once, and never returned. What was the consequence? Nothing at all. Did she fall into a bad state of health? No. Did she die? No. She continued in good health for thirty years more, and died of old age. Instead of suffering any injury from the cessation of the effusion, she was happier and more comfortable without it."

Now to us it appears absurd to infer, that because the health of a female does not suffer by the non-performance of a certain function before the organ which is to perform that function is completely evolved, nor after the period designed by nature for its duration, therefore the health cannot be influenced by its due or imperfect performance during the usual time of its continuance: because she suffers no injury from the cessation of the effusion at the period nature designed it to cease, she cannot suffer any by its interruption during the time nature intended it to flow. Besides, is Mr. Fogo certain that those "who are happier and more comfortable" on its total cessation, are not precisely those who have experienced the most regular performance of it during the natural period of its continuance?

His remarks, Mr. F. tells us, were called forth by a paper in the last Number of the Edinburgh Journal, wherein the Author endeavours to show the probability of Amenorrhœa being a frequent cause of morbid affections of the lungs, in certain states of predisposition. This connexion Mr. F. denies; and as Dr. Shearman illustrated his general principles by a particular case, Mr. Fogo attempts to explain the phenomena of that case by different and opposite principles. In doing this it was surely incumbent upon Mr. F. to admit the statement given in that paper, and to ground his reasonings upon the same data. Now Dr. S. distinctly says, "a young woman, *in pretty good health*, experienced an obstruction of the menses in *consequence of cold; from this time* her health gradually declined, she became languid and weak, &c." It suits Mr. F. however, to invert the order of occurrence of these symptoms, and and because the mesenteric glands were found diseased after death, to infer a "probability next to a certainty, that disease in these most indispensably necessary organs was the *original* cause of the emaciation, debility, and *consequently* of amenorrhœa." Putting aside the particular case, for Dr. S. does not appear to *found* his general principles upon that case, only to illustrate them by it, and taking a broader ground, let the parties join issue upon the facts. Dr. S. says, "No occurrence is more common than the attack of cough, pain in the side, and difficulty of breathing in females, soon *after* the obstruction of the menses, and upon their recurrence all these symptoms going off." If this be true, then the position of Dr. S. that amenorrhœa sometimes proves a cause of disease *may* be true also; it is not *necessarily* false. Mr. F. says, amenorrhœa or chlorosis, "never were, nor ever can be, the cause of bad health," he never regards them as any thing more than symptoms, or effects of some *previous* disease. "When no remarkable evacuations have taken place, the cause will very possibly be discovered in some derangement of the organs which contribute to the indispensable processes of digestion, chylification, sanguification, and nutrition. When these are in a state of health, there *never will be* amenorrhœa or chlorosis met with. When these organs are in a state of health, the whole system is supplied with what is necessary. The uterus will partake, and *undoubtedly* its functions will be carried on." If cases ever occur in which, during the perfect performance of all these four functions, the menses are *suddenly* suppressed by some accidental cause, as from taking cold; if while "the whole system is supplied with what is necessary, and the uterus is partaking," the functions of the uterus are impeded, *and are not* carried on, then Mr. F's axiom is *not true*, it must necessarily be false. Experience alone can determine this question, and to the experience of our Readers we leave it. We certainly have fancied that we have seen strong healthy girls, with their vessels full of blood, and the uterus partaking, in whom the menses have, notwithstanding, been obstructed, and we have fancied that we have seen paleness, emaciation and debility *follow* this obstruction. If all this should have occurred in persons of certain predispositions, and there had *at length* taken place affections of the lungs, or of the mesenteric glands, and death had followed, we should not have denied that these last affections were *accessory* to the death of the patient, although we should still have maintained the *original cause* to have been the menstrual obstruction.

The INQUIRER, No. XVIII.—Many Practitioners think that the use of mercury has become much too fashionable, and that its indiscriminate employment has been productive of no little mischief. Indeed, some of those who were formerly advocates for its frequent exhibition, now think it necessary to endeavour to check the prevailing rage for this metal. The Inquirer is of opinion, that there are solid objections to so constant an employment of it as has been adopted of late; and when one of its preparations, calomel, lomel, has been given in fevers, and recovery has followed, he is inclined to attribute the good effects to its purgative qualities alone.

"A boy, 14 years of age, had his hand torn by a wheel. No material inconvenience was felt for ten days after the accident; he then began to complain of stiffness about his neck and chin; his jaw became locked; and this symptom was followed by pain at the sternum, spasmodic contraction of the abdominal muscles, and rigid contraction of all the muscles of his extremities and back; a quick and small pulse, and great anxiety in his countenance. The wound in his hand was not inflamed or painful, but went on healing, as if the system had not been at all deranged. Affusion of cold water was applied, without any relief to the symptoms; calomel and opium were administered internally, in large doses, for several days; but the disease kept increasing, though not in a violent degree. The warm bath was then ordered, and half an ounce of strong mercurial ointment was rubbed over the abdomen, and on the inside of the thighs and arms, after coming from the bath, every night and morning. These remedies were carefully administered for four days, with manifest advantage. No salivation was produced by the mercury; and no apparent effect, except violent diarrhœa, which began on the second day after employing mercurial frictions; and this was allowed to continue unchecked for several days, as the most urgent symptoms gradually abated, while the bowels were so much relaxed. When the symptoms of tetanus had subsided, some restringent medicine was given; and at the end of three weeks from the commencement of the trismus, the boy was perfectly recovered.

"The recovery of a patient from tetanus induced by a lacerated wound, is not an every-day occurrence; it excites the inquiry, what cured him? The *mercury*, says one physician,—the *purgative*, says another,—the *warm bath*, says a third,—and a fourth will perhaps honestly confess that he is ignorant of the cause, he only knows the fact, that the patient recovered by employing active remedies, when, in all probability, he would have died if he had been left unassisted."

